# Infections in residents of long- term care facilities in southern Poland, 12-month surveillance preliminary results

**DOI:** 10.1017/ash.2024.299

**Published:** 2024-09-16

**Authors:** Izabella Bylica, Jadwiga Wójkowska-Mach, Zofia Gniadek, Karolina Gutkowska

**Affiliations:** Doctoral School of Medical and Health Sciences, Jagiellonian University Medical College, Krakow, Poland; Jagiellonian University Medical School

## Abstract

Long-Term Care Facility (LTCF) residents are particularly vulnerable to infections due to factors such as advanced age, co-morbidities, and regular medication use. Data from American and European sources indicate an anticipated occurrence rate of 2 to 11 infections per 1000 patient-days (pds) in LTCFs. The incidence rate of Clostridioides difficile infections (CDI) is reported at 0.52 per 10,000 resident days. The research objectives aimed to assess infection epidemiology in Polish LTCFs. An observational prospective study was conducted on residents from five LTCFs (2 residential homes and 3 nursing homes) in southern Poland between September 2022 and September 2023, utilizing the definition from the pan-European HALT study. The study received approval from the Bioethics Committee of the JU (1072.6120.73.2022) and was funded by the Polish NCN grant No.2021/41/B/NZ6/00749. CDI was defined by positive toxins A and B enzyme immunoassays (EIA) and positive glutamate dehydrogenase (DHA) EIA. Results from the study, involving 250 residents, revealed 157 cases of Healthcare-Associated Infections (HAIs) excluding gastrointestinal and CDI, with an incidence rate of 1.97/1000 pds. Lower respiratory tract infections dominated with 77 cases, including 36 pneumonia cases (47%). Additionally, 25 cases of gastrointestinal infections were reported, including only 7 CDI cases, resulting in an incidence rate of 0.88 CDI per 10,000 pds. Norovirus was detected in only one case, while the microbiological results were negative in the remaining cases. The incidence rate among Polish LTCF residents was lower than expected, contrasting with the CDI incidence that aligned with other research findings. Notably, the etiology of diarrhea remained undetermined in 68% of cases.

